# Detection of artificial pulmonary lung nodules in ultralow-dose CT using an ex vivo lung phantom

**DOI:** 10.1371/journal.pone.0190501

**Published:** 2018-01-03

**Authors:** Caroline Alexandra Burgard, Thomas Gaass, Madeleine Bonert, David Bondesson, Natalie Thaens, Maximilian Ferdinand Reiser, Julien Dinkel

**Affiliations:** 1 Department of Clinical Radiology, University Hospital Munich, Ludwig-Maximilians-University, Munich, Germany; 2 Comprehensive Pneumology Center, German Center for Lung Research, Munich, Germany; 3 German Cancer Research Center, Division of Medical Physics in Radiology, Heidelberg, Germany; Northwestern University Feinberg School of Medicine, UNITED STATES

## Abstract

**Objectives:**

To assess the image quality of 3 different ultralow-dose CT protocols on pulmonary nodule depiction in a ventilated ex vivo-system.

**Materials and methods:**

Four porcine lungs were inflated inside a dedicated chest phantom and prepared with n = 195 artificial nodules (0.5–1 mL). The artificial chest wall was filled with water to simulate the absorption of a human chest. Images were acquired with a 2x192-row detector CT using low-dose (reference protocol with a tube voltage of 120 kV) and 3 different ULD protocols (respective effective doses: 1mSv and 0.1mSv). A different tube voltage was used for each ULD protocol: 70kV, 100kV with tin filter (100kV_Sn) and 150kV with tin filter (150kV_Sn). Nodule delineation was assessed by two observers (scores 1–5, 1 = unsure, 5 = high confidence).

**Results:**

The diameter of the 195 detected artificial nodules ranged from 0.9–21.5 mm (mean 7.84 mm ± 5.31). The best ULD scores were achieved using 100kV_Sn and 70 kV ULD protocols (4.14 and 4.06 respectively). Both protocols were not significantly different (p = 0.244).

The mean score of 3.78 in ULD 150kV_Sn was significantly lower compared to the 100kV_Sn ULD protocol (p = 0.008).

**Conclusion:**

The results of this experiment, conducted in a realistic setting show the feasibility of ultralow-dose CT for the detection of pulmonary nodules.

## Introduction

Lung cancer is the leading cause of cancer deaths in men, and the second leading cause of cancer deaths in women after breast cancer [1, 2]. Curative treatment in the form of surgical resection offers the best chance of survival. Therefore, detection of early-stage cancer by computed tomography imaging is indispensable for a successful treatment. The analysis conducted within a large multicenter trial (National Lung Screening Trial [3, 4]) showed a 20% reduction in mortality rate of a high risk population undergoing annual lung cancer screening with low dose computed tomography (LDCT). However, debates about an assumed radiation-associated risk of cancer development from ionizing radiation continue to limit the widespread application of LDCT screening. Due to repetitive CT (computed tomography) examinations, patients, who receive an annual LDCT (effective dose of 5.2 mGy or approximately 1 mSv) from the age of 50–75 years showed an additional risk of 1.8% (95% CI 0.5–5.5) for lung cancer development [5]. Thus, following the ALARA (as low as reasonably achievable) principle, the radiation dose of each computed tomography study should be reduced to a level that is “as low as reasonably achievable”. Various strategies have been developed for lowering the radiation dose of CT without influencing the signal-to-noise-ratio, including lowering the tube voltage and/or the tube current, noise reduction filters, iterative reconstruction selective in-plane shielding or automated exposure control [6–10].

Recently, ultralow-dose scans with doses close to conventional chest radiography (approximately 0.3 mSv) [1, 2, 11, 12] can be performed by third-generation dual-source CTs by using a tin filter (TF) that is mounted in front of both x-Ray tubes. The so called spectral-shaping of the high-kVp beam leads to a more efficient x-Ray beam which results in a reduction of the radiation dose [13]. Furthermore, a new generation of IR was developed in third-generation dual-source CT, so called advanced model-based IR (ADMIRE), which allows for a decrease of the radiation exposure by retrospectively compensating for increased image noise.

Most of the previous studies on detection of pulmonary nodules in ULD CT were performed in anthropomorphic chest phantoms or performed in second-generation dual-source CT with outdated IR versions [7, 14–16]. To our knowledge, there is little data available regarding the image quality and the diagnostic confidence of ULD chest CT and reconstructions using ADMIRE for the detection of pulmonary nodules in a realistic phantom model. Consequently, we created artificial nodules of clinically relevant sizes in an *ex vivo* lung phantom containing porcine lung explants. This experimental set up allows for repeated scanning using different combinations of tube potential and tube current-time-product in ULD chest CT. The purpose of this study was to evaluate the feasibility of ULD CT as early-stage lung cancer detection method using ULD CT protocols with 3 different tube potentials for the detection of pulmonary nodules in an *ex vivo* lung phantom.

## Materials and methods

### Ex vivo lung phantom

For this study, a double-walled chest phantom (Artichest®, PROdesign GmbH, Heilikreuzsteinach, Germany) was employed as previously described [17, 18]. The system comprises two copolymer containers with a 2–5 cm space between the inner and outer shell and an artificial diaphragm both of which were filled with pure water to simulate the attenuation of the chest wall and upper abdomen of an overweighed patient with a body weight about 100 kg as previously described [19]. Four freshly excised porcine lungs (including the heart) were subsequently placed in the bottom casing of phantom container and connected to room atmosphere via a 7.5 mm tracheal tube (Portex; SIMS Portex Ltd., Hythe, Kent, UK) through a dedicated outlet. In order to inflate the lungs for nodule placement the tracheal tube was connected to a resuscitation bag. After the nodule insertion the phantom casing was hermitically sealed and the lungs were passively inflated by continuous evacuation of the artificial pleura space to -2 –-3 x 103 Pa. All heart-lung explants of mature pigs were obtained by a local slaughterhouse (Muenchner Schlachthof Betriebs GmbH, Zenettistraße 10, 80337 Munich, Germany) with special attention to intact pleura. Institutional Review Board approval or animal research ethics committee approval was not required because no human being participated in this study and no animal was euthanized for the particular purpose of this study. No anesthesia, euthanasia, or any kind of animal sacrifice was part of this study.

### Preparation of artificial lung nodules

Lung nodules were simulated using agar (Roth Chemie GmbH, Karlsruhe, Germany) gel, prepared from a 3% agar-water solution. During continuous inflation via the resuscitation bag 20–30 injections of 0.5–1.0 mL hand-warm agar gel were carried using a 5 mL syringe (BD Discardit®II, Becton Dickinson, Heidelberg, Germany) and a 20 G cannula (Sterican®, B. Braun Melsungen AG, Melsungen, Germany). Injections were distributed over the whole lung at depths of 3–6 cm, resulting in artificial nodules with a mean density of 25 Hounsfield Units (HU) at 100 kV.

### CT scan settings

All multi-detector computed tomography acquisitions were performed on a third-generation dual-source CT machine (SOMATOM Force, Siemens Healthcare, Erlangen, Germany). Each prepared lung was scanned using identical parameters: collimation: 0.6 mm, number of slices: 192, gantry rotation time: 0.25–0.5 s, pitch: 0.5–1.2, scan time 3–10 s. A standard dose protocol was carried out as reference at a tube voltage of 120 kV, resulting in a CTDI_vol_ of 1.8 (approx. 1 mSv effective radiation dose). The ULD protocol was acquired at tube voltages of 70kV, 100Sn (tin) kV and 150Sn kV. To obtain a defined radiation dose level with a DLP of 7.5 mGy*cm (approx. 0.1 mSv simulated effective radiation dose), reference tube current-time products (mAs) were adjusted by Siemens automatic exposure control system (CARE Dose 4D) (Table 1). Images of every protocol-including the reference standard protocol with 120 kV were reconstructed using advanced-modeled iterative reconstruction (ADMIRE) at level 3.

**Table 1:** CT acquisition protocols.

Name of protocol	Reference standard protocol	ULD protocol 1	ULD protocol 2	ULD protocol 3
**Tube voltage (kV)**	120 kV	70 kV	100 kV Sn	150 kV Sn
**CTDI**_**vol**_	1.8	0.18	0.18	0.18
**Mean of tube current- time product (mAs)**	27	4	24	4
**Pitch**	0.9	0.7	0.5	1.2

Iterative reconstruction was performed using a commercially available algorithm (ADMIRE®, Siemens Healthcare, Erlangen, Germany) at level 3. ADMIRE is a model-based iterative algorithm. Level 3 stands for the number of iteration cycles and complies with standard image quality. The reconstruction resulted in data sets with a slice thickness of 0.75 mm, a slice increment of 0.6 mm, using a sharp tissue kernel (Br69), a matrix of 512 x 512 pixels and a field of view of 330 mm.

### Evaluation of image quality

Two blinded readers (radiologists, 2 years and 10 years of experience, respectively) evaluated the diagnostic confidence for each nodule on a modified 5-point Likert scale: 1 ― non-diagnostic quality, strong artifacts, insufficient for diagnostic purposes score; 2 ― severe blurring with uncertainty about the evaluation; 3 ― moderate blurring with restricted assessment; 4 ― slight blurring with unrestricted diagnostic image evaluation possible; 5 ― excellent image quality, no artifacts. The Siemens Syngo CT Oncology®software tool (Syngo MultiModality Workspace VE36A, Siemens Healthcare, Erlangen, Germany) was employed to measure the maximum diameter of the nodules and evaluating the image quality in coronal, axial and sagittal multiplanar reformations (MPR). For exact evaluation of the artificial lung nodules, the CT scans were separated in three different parts (upper, middle and lower part) to depict differences in image quality and artifacts caused by characteristics of the lung phantom, e.g. the artificial diaphragm.

### Statistical analysis

All data were recorded in a dedicated database (Excel®2010, Miscrosoft Corp., Redmond, USA) and analyzed using SAS for Windows® (Version 9.4, SAS Institute Inc., Cary, NC, USA) and BiAS. for Windows®(Version 11.02, Epsilon, Frankfurt am Main, Germany) software. Differences in the score obtained for two different ULD protocols were tested for statistical significance using a Wilcoxon–matched–pairs test. To measure the interrater reliability between the scores of the two observers, Cohen’s (weighted) Kappa was determined. To measure the interrater reliability between the scores of the two observers, Cohen’s (linearly weighted) Kappa was calculated. A weighted Kappa of 0.69 or higher was rated as good interrater reliability. For all tests, a p-value of <0.05 or <0.05/m (number of tests) corrected with Bonferroni’s method was considered as statistically significant. In addition, proportions of agreement between the two readers were calculated.

## Results

### Characteristics of artificial nodules

Overall, 214 artificial nodules were created. All nodules with good demarcation, round shape, and a solid character (n = 195) were judged to be typical for small solid nodules such as metastases or lung cancer. Lesions with poorly defined boundaries, a part-solid aspect, or distinct draining of mixture into a bronchus, blood vessel, or the injection pathway (n = 19) were excluded from evaluation. Mean diameter of the artificial nodules were 7.84 mm ± 5.31 (range 0.9–21.5 mm).

### Assessment of diagnostic confidence

**Fig 1:**
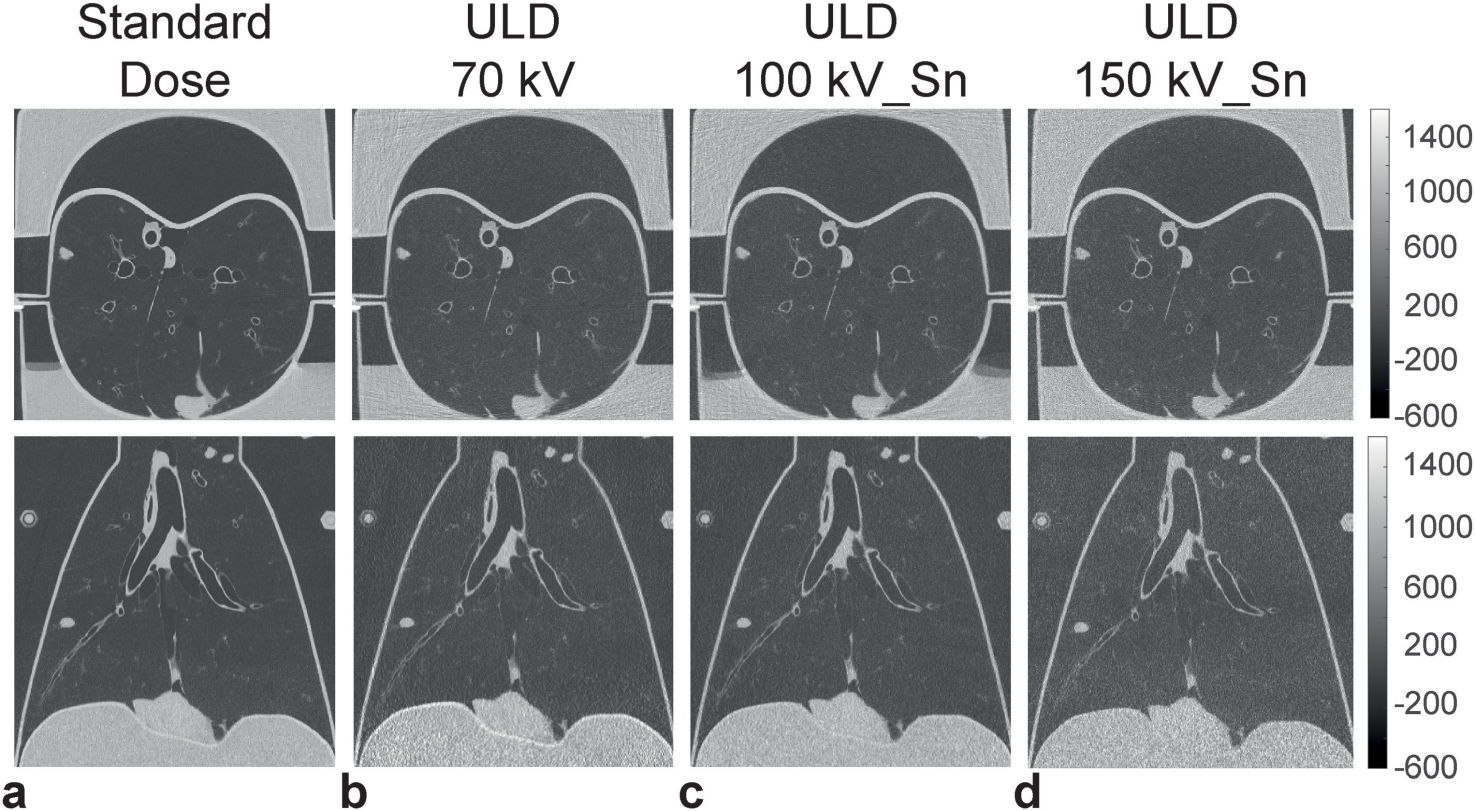
Representative axial and coronary reconstructed images of the standard dose protocol (a) and the carried out ULD protocols with 70 kV (b), 100 kV_Sn (c) and 150 kV_Sn (d).

**Fig 2:**
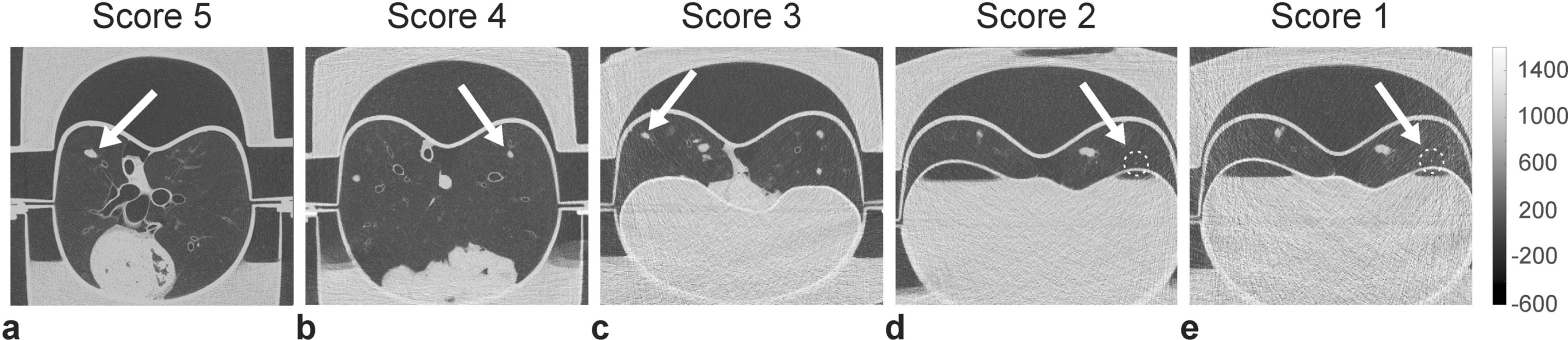
Representative axial reconstructed images, depicting each score (1 = non diagnostic image quality, 2 = severe blurring, 3 = moderate blurring, 4 = slight blurring, 5 = excellent image quality), exemplary. a) Reference protocol (120 kV), b) ULD protocol 2 (100 kV Sn), c) ULD protocol 1 (70 kV), d) ULD protocol 2 (100, kV Sn), e) ULD protocol 1 (70 kV).

Representative images for the reference protocol and different ULD CT scans are displayed in Fig 1. Exemplary scores (1–5) of different nodule are provided within Fig 2. The subjective impression of the image quality was excellent in images of the reference CT protocol and of all three ULD CT protocols as well. In the reference protocol every artificial nodule was rated with an excellent score of 5 due to excellent image quality and missing artifacts. With a score of 4, the ULD CT protocol with 100 kV Sn showed the best image quality. Close to the artificial diaphragm, some of the nodules were evaluated with a score of 2 or 3 in the 70 kV ULD protocol due to beam hardening artifacts.

**Fig 3:**
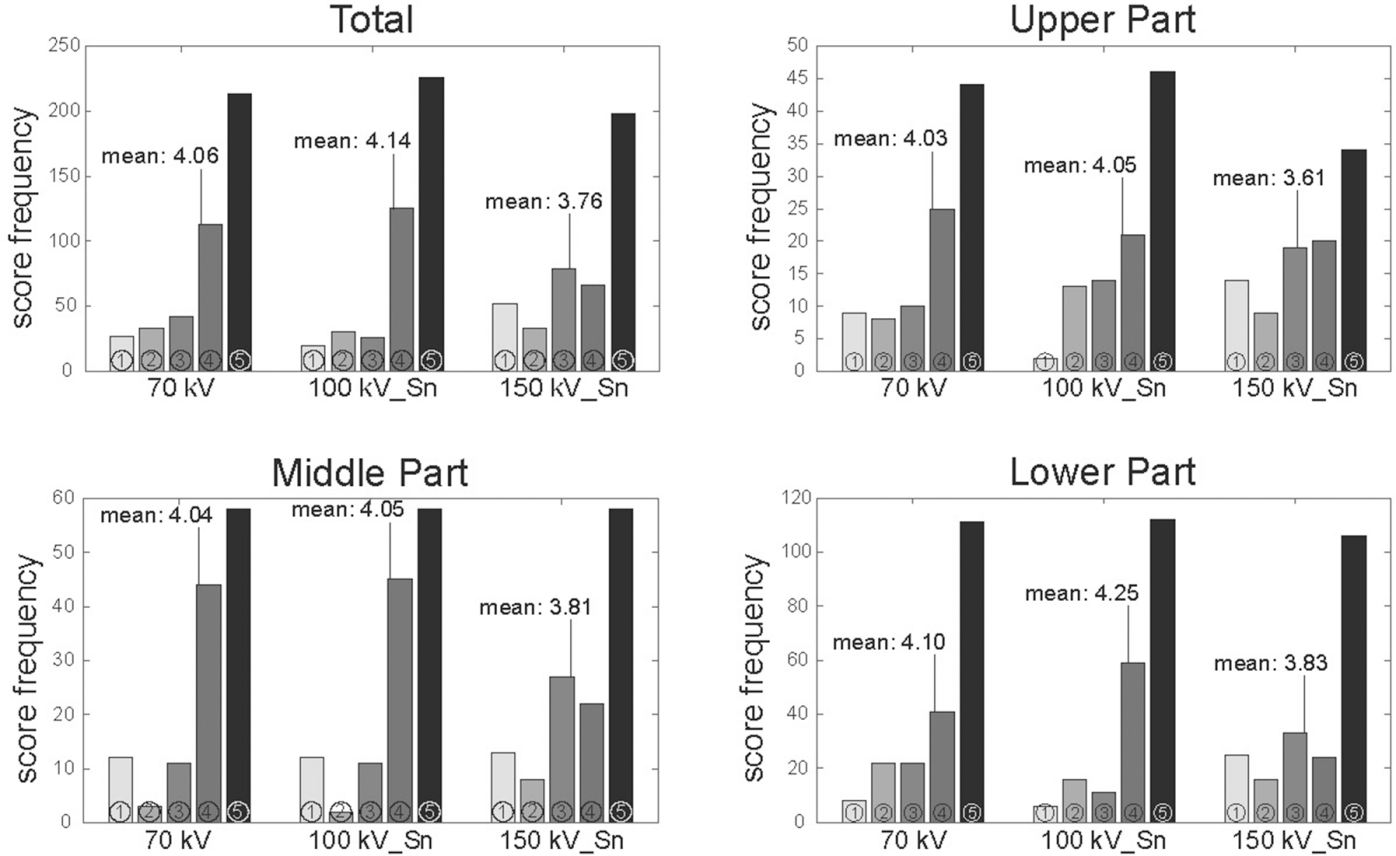
Score frequency of diagnostic confidence of lung nodule detection in different ULD CT protocols (70 kV, 100 kV_Sn and 150 kV_Sn) in comparison: Total lung phantom (n = 195 nodules), upper part (n = 46 nodules), middle part (n = 74 nodules) and lower part (n = 75 nodules). In the reference protocol with 120 kV every artificial nodule was evaluated with the highest score of 5 and therefore the mean was 5.

The overall diagnostic confidence of pulmonary nodule detection was rated best by the two blinded readers in the ULD CT protocol with 100 kV Sn with a score of 4.14 and in the 70 kV ULD protocol with a score of 4.06 (Fig 3- Total). The lowest mean score of 3.78 was achieved using the 150 kV Sn due to the lowest rated score of 1 that was assigned to 52 artificial nodules with strong artifacts and severe blurring of the nodules edges. For ULD CT protocols, the confidence of nodule detection was not significantly higher in images acquired at a tube voltage of 100 kV Sn compared with 70 kV (p = 0.244). Compared to ULD CT protocol with 100 kV Sn, the images acquired with 150 kV Sn showed a significant lower diagnostic confidence of nodule detection (p = 0.008). Images acquired with the 70 kV ULD CT protocol showed also higher scores compared to the 150 kV ULD CT protocol, but this did not reach statistical significance (p = 0.08). The median score for ULD protocol 1 with 70 kV was 4.5, for ULD protocol 2 with 100 kV Sn 5.0, and for the ULD protocol 3 with 150 kV Sn 4.0. The reference protocol with 120 kV had a median of 5 as well.

When separating the lung phantom in three different parts (upper, middle and lower third), the best scores for confidence of nodule detection were still achieved using ULD CT protocols with a tube voltage of 100 kV Sn and 70 kV (Fig 3 Upper, Middle and Lower Part). Again, both protocols didn’t show a significant difference (upper third: p = 0.439, middle third: 0.402, lower third: p = 0.107). Like presented before, the lowest score for diagnostic confidence was reached for ULD CT protocols with 150 kV Sn, irrespective of the location of the nodules in the lung phantom. The difference of the scores for diagnostic confidence were significant for the 70 kV and 150 kV Sn ULD protocols in the upper part of the lung phantom (p<0.001) but not in the middle or lower part (p = 0.066 and p = 0.141, respectively). The 100 kV Sn ULD protocols showed significant differences in the scores to the 150 kV Sn protocols in the upper and lower third of the lung phantom (p = 0.016 (corrected with Bonferroni’s method) and p<0.001, respectively). In the middle part, the difference between the 100 kV Sn and 150 kV Sn did not reach statistical significance (p = 0.021).

### Interrater reliability and agreement for diagnostic confidence

Overall, a good to excellent interrater reliability for the diagnostic confidence in detection of artificial pulmonary nodules in ULD CT protocols was achieved with a Cohen’s weighted kappa ranging from 0.696 to 0.882 (Table 2). The best interrater reliability for diagnostic confidence in total lung phantom was achieved in the 100 kV Sn ULD CT protocol with a Cohen’s weighted kappa of 0.798, the worst in 70 kV ULD CT protocol with 0.775. In 70 kV and 150 kV Sn ULD CT protocols, an exact agreement between both observers of 87.7% of nodules was reached, whereas in 100 kV Sn ULD CT protocol the interobserver agreement accounted for 89.7%.

**Table 2:** Interrater reliability (Cohen’s weighted kappa) of diagnostic confidence of lung nodule detection in different ULD CT protocols in comparison.

ULD CT Protocols	Total Lung phantom	Different Regions of Lung phantom		
		Upper Part	Middle Part	Lower Part
**100 kV Sn**	0.798	0.696	0.881	0.790
**150 kV Sn**	0.782	0.701	0.823	0.795
**70 kV**	0.775	0.757	0.882	0.710

For the upper part of the lung phantom, the best interrater reliability was in 70 kV ULD CT protocol with a Cohen’s weighted kappa of 0.757, the worst in the 100 kV Sn ULD CT protocol with 0.696. In the middle part of the lung phantom, interrater reliability showed the best results in the 70 kV ULD CT protocol with a Cohen’s weighted kappa of 0.882, and the worst in 150 kV ULD CT protocol with 0.823. Cohen’s weighted kappa was best in the 150 kV Sn ULD CT protocol (0.795) in the lower part of the lung phantom meaning the best interrater reliability, and worst in 70 kV ULD CT protocol with 0.710.

For the upper, middle and lower part of the phantom, the interrater agreement was best in 100 kV Sn ULD CT protocol with 89.8%, 89.9% and 89.7%, respectively. In 70 kV ULD CT protocol, the interobserver agreement was best in the middle part of the phantom with 87.9%, worst in the lower part with 87.7% and 87.8%. The best rate of interobserver agreement was in the middle part of the phantom with 150 kV Sn ULD CT protocol with 87.9%, the worst in the upper part with 87.6% and 87.8% in the lower part.

## Discussion

In this study, we assessed the value and diagnostic confidence of ultralow-dose CT for detection of artificial pulmonary lung nodules acquired by a third-generation dual-source CT using CARE Dose 4D and advanced-modeled iterative reconstruction (ADMIRE). For this purpose, a ventilated ex vivo lung phantom was scanned containing artificial solid nodules of various sizes at random distribution resulting in an effective dose of 1/10th of the low dose value. Our results indicated that image quality remains at a high level by using ultralow-dose scan protocols, and diagnostic confidence of artificial pulmonary nodule detection as well as the interobserver reliability and agreement were best when using a protocol with 100 kV tube voltage with tin filtration and the newest generation of iterative reconstruction (ADMIRE). To the best of our knowledge, this is the first study to compare diagnostic confidence for detection of pulmonary nodules in a realistic ex vivo lung phantom simulating an overweighted patient in ULD CT scan protocols. The experimental setup allowed for scanning identical anatomical conditions repeatedly at multiple exposure settings from standard to ultralow-dose and with different tube voltages. In previous studies [15,16], the nodule density, lung parenchyma density and noise in the employed chest phantom were similar to images of heavy smokers participating in lung cancer screening, which results in a more realistic setting compared to other anthropomorphic chest phantoms. In these previous studies, the ex vivo lung phantom was acquired with a second generation dual source CT with low dose protocol and reconstructed by FBP and second generation IR (SAFIRE) [15,16].

Alkadhi et al. evaluated the image quality and sensitivity of ULD CT using third generation dual source CT with tube voltages similar to our study (70kV, 100 kv Sn and 150 kV Sn) [12]. As opposed to our study setting they used an anthropomorphic chest phantom simulating an intermediate-sized adult. A common critic of low dose studies on phantom is that they represent an ideal patient instead of an overweight patient with 100 kg like in our study for a more realistic experimental setting.

In accordance with the study of Alkadhi et al [12], the diagnostic confidence of lung nodule detection in our study was best in images acquired with the ULD scan protocol at a tube voltage of 100 kV with tin filter. Unlike Alkadhi et al, we found the lowest diagnostic confidence of artificial lung nodule detection in ULD protocols with 150 kV Sn compared to the results of the study of Alkadhi which rated the ULD scan protocol with 70 kV of limited value for non-enhanced chest CT. These differences can be explained by the fact that a more realistic lung phantom was used in our study compared to the anthropormorphic chest phantom used by Alkadhi. Despite the increased attenuation due to the double chest wall, the quality of images performed with a 70 and 100 kV spectrum was much better than with the beam hardened 150kV (tin filter).

The electron density of lung parenchyma is probably so low that high energetic, beam hardened photons passes through the lung parenchyma, which results a dramatic loss of contrast. In addition, Compton effects due to the more energetic photons are decreasing the quality and the contrast between lung parenchyma and artificial nodule further.

Martini et al. tested ULD CT protocol with 100 kV and tin filter with third generation dual source CT with an anthropomorphic chest phantom that simulated normal weighted and obese patients, respectively [20, 21]. However, they just assessed the sensitivity of nodule detection and the general image quality and did not compare different ULD CT protocols among each other.

Another prior lung phantom study showed the feasibility of ultralow-dose CT in lung cancer screening [15] with nearly the same high sensitivity in lung nodule detection compared to the standard dose. But the images were acquired at a second generation dual-source CT scanner without circuit detector and spectral shaping and only with a tube voltage of 80kV. The utilized anthropomorphic chest phantom presented the characteristics of a 70-kg male individual and lacked of realistic lung parenchyma. Nevertheless, an anthropomorphic chest phantom has some advantages like the reproducible experimental conditions. The disadvantage of the ex vivo lung phantom used in our study are the sensitivity of the system on the first hand because it has to be sealed and stable to create a vacuum for lung inflation. That can just be realized by a double wall at the expense of the attenuation. On the other hand, the double wall is not able to exactly simulate a thoracic wall with ribs and the experimental setting is dependent on the quality and condition of the inflated pig’s lung.

Recently, Sui et al. [14] reported a high confidence for evaluating lung nodules in ultralow -dose CT in patients at 0.13 mSv with a tube voltage of 80 kV and a tube current-time of 4 mAs and reconstructed by second generation IR (SAFIRE) and FBP, respectively. The use of SAFIRE allows for a reduction of radiation dose by approximately 65% without the loss of diagnostic information in low-dose chest CT [22].

The third-generation dual-source CT machine which was used in our study, included the newest iterative reconstruction technique by Siemens, called ADMIRE. This technique includes statistical data modeling in the raw data domain and combines it with model-based noise detection in the image domain by using an iterative approach. ADMIRE shows an image noise reduction of 50% compared to SAFIRE in ultralow-dose chest CT [12]. Additional by adding a tin filter, the shape of the applied energy spectrum is significantly modified, and less efficient energy spectra are removed. Combining the newest third-generation iterative reconstruction technique and spectral shaping of the high kV-beam, allows for dose reduction and leads to ultralow-dose CT protocols with dose levels close to those of conventional chest x-Ray like in our study without decreasing image quality and diagnostic confidence, and despite a chest phantom presenting the characteristics of a 100-kg male individual.

Our study has some limitations. First, we have to acknowledge the inherent limitations of a phantom study. Although the attenuation of a porcine lung is close to a human lung and the density of the artificial lung nodules are similar to realistic malign lung nodules, it can never substitute a real patient with individual body constitutions that have influence on the effective dose. In addition, our ex vivo lung phantom missed physiological motion (cardiac or residual respiratory motion). However, repetitive scanning with various CT protocols precludes application in humans for ethical reasons and the gold standard is missing in vivo. Second, no other tube voltages were applied like 80, 110 or 130 kV, and there was no comparison of images reconstructed by different ADMIRE strength levels. Furthermore, we used different pitch levels for each ULD CT protocol, which could have influence on the image quality, though it is negligible for a multi-line CT machine (192 lines). Finally, we examined ultra-low dose protocols and reconstruction techniques for solid, but not for ground-glass or part-solid nodules like in other studies [7, 21].

In conclusion, our study suggests that detecting pulmonary nodules in ultralow-dose chest CT is feasible and the image quality and diagnostic confidence were excellent in a dedicated protocol with 100 kV with spectral shaping and when using third-generation iterative reconstructions techniques. Future studies with real patients will have to assess the feasibility of ULD CT screening protocols in intention to further reduce the effective radiation dose for patients.

## Supporting information

S1 Dataset Nodule sizes and scores of diagnostic confidence of each nodule in reference protocol and three different ULD CT protocols. (XLSX)
